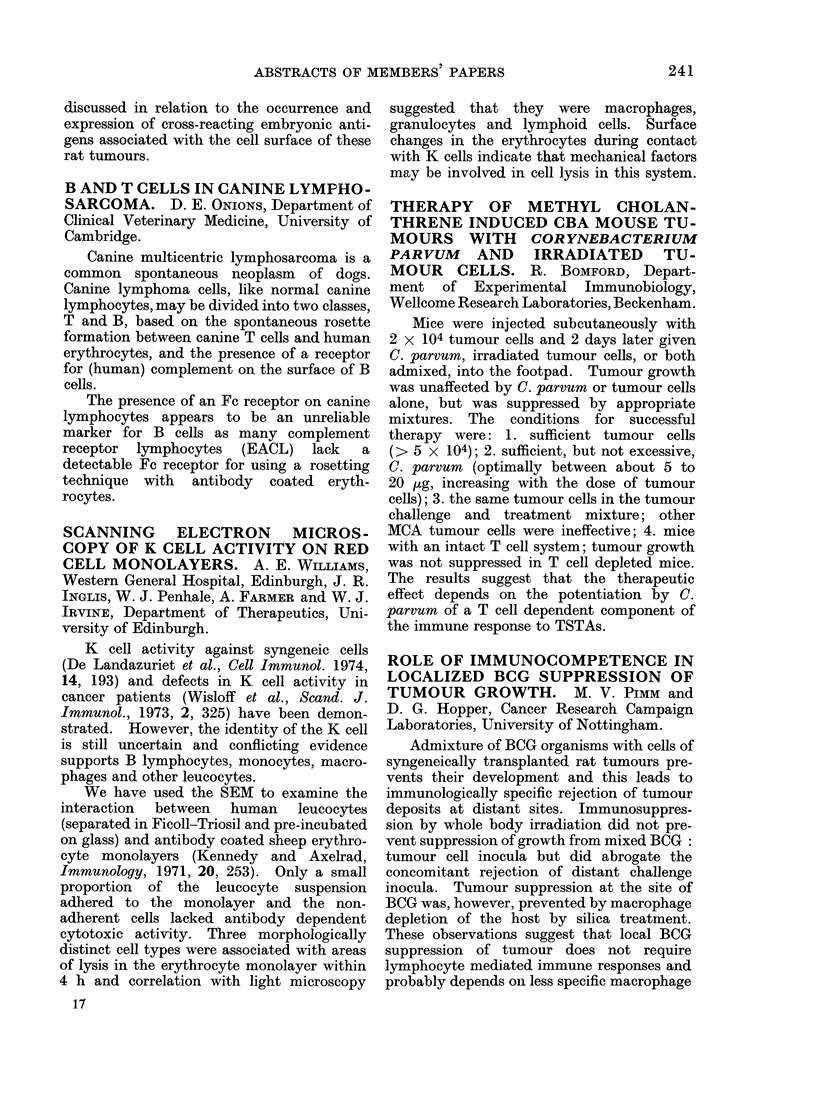# Proceedings: Scanning electron microscopy of K cell activity on red cell monolayers.

**DOI:** 10.1038/bjc.1975.158

**Published:** 1975-08

**Authors:** A. E. Williams, J. R. Inglis, W. J. Penhale, A. Farmer, W. J. Irvine


**SCANNING ELECTRON MICROSCOPY OF K CELL ACTIVITY ON RED CELL MONOLAYERS.** A. E. Williams, Western General Hospital, Edinburgh, J. R. Inglis, W. J. Penhale, A. Farmer and W. J. Irvine, Department of Therapeutics, University of Edinburgh.

K cell activity against syngeneic cells (De Landazuriet *et al.*, *Cell Immunol.* 1974, **14**, 193) and defects in K cell activity in cancer patients (Wisloff *et al.*, *Scand. J. Immunol.*, 1973, **2**, 325) have been demonstrated. However, the identity of the K cell is still uncertain and conflicting evidence supports B lymphocytes, monocytes, macrophages and other leucocytes.

We have used the SEM to examine the interaction between human leucocytes (separated in Ficoll–Triosil and pre-incubated on glass) and antibody coated sheep erythrocyte monolayers (Kennedy and Axelrad, *Immunology*, 1971, **20**, 253). Only a small proportion of the leucocyte suspension adhered to the monolayer and the non-adherent cells lacked antibody dependent cytotoxic activity. Three morphologically distinct cell types were associated with areas of lysis in the erythrocyte monolayer within 4 h and correlation with light microscopy suggested that they were macrophages, granulocytes and lymphoid cells. Surface changes in the erythrocytes during contact with K cells indicate that mechanical factors may be involved in cell lysis in this system.